# Review of Three-Dimensional Human-Computer Interaction with Focus on the Leap Motion Controller

**DOI:** 10.3390/s18072194

**Published:** 2018-07-07

**Authors:** Daniel Bachmann, Frank Weichert, Gerhard Rinkenauer

**Affiliations:** 1 Department of Computer Science VII, TU Dortmund University, 44221 Dortmund, Germany; daniel.bachmann@tu-dortmund.de; 2 Leibniz Research Centre for Working Environment and Human Factors, 44139 Dortmund, Germany; rinkenauer@ifado.de

**Keywords:** human-computer interaction, contact-free input devices, three-dimensional interaction, natural user interfaces, leap motion controller

## Abstract

Modern hardware and software development has led to an evolution of user interfaces from command-line to natural user interfaces for virtual immersive environments. Gestures imitating real-world interaction tasks increasingly replace classical two-dimensional interfaces based on Windows/Icons/Menus/Pointers (WIMP) or touch metaphors. Thus, the purpose of this paper is to survey the state-of-the-art Human-Computer Interaction (HCI) techniques with a focus on the special field of three-dimensional interaction. This includes an overview of currently available interaction devices, their applications of usage and underlying methods for gesture design and recognition. Focus is on interfaces based on the Leap Motion Controller (LMC) and corresponding methods of gesture design and recognition. Further, a review of evaluation methods for the proposed natural user interfaces is given.

## 1. Introduction

Traditional two-dimensional User Interfaces (UIs) are familiar to most users. This includes devices like mouse and keyboard as input and Liquid Crystal Display (LCD) or multi-touch displays as output systems. However, modern software and hardware development has led to systems that are often not suitable for keyboard input and standard display-based output technologies. Especially when (mobile) virtual environments are designed, developers as well as users search for input devices that lead to a more intuitive interaction experience. Krueger et al. [1] stated early that, as greater parts of the population get in touch with computer systems, interaction systems have to move closer to the process used to accomplish the same interactions in the physical world. This approach is described by the term natural interaction. Devices claiming to help produce this experience are referred to as natural interaction devices and build the foundation of so-called Natural User Interfaces (NUIs) (cf. Section 2). NUIs are special types of User Interfaces (UIs), that support interactivity by analysing and recognising users and their behaviour. Input is based on a wide range of interaction devices, which in turn are based on, for example, cameras, depth sensors or haptic or tactile feedback (cf. Section 2.2). However, with new devices and interfaces, new (standardised) metaphors and interaction methodologies have to be designed and evaluated.

The main objective of this work is the review of NUI interaction design based on three-dimensional input devices. The main contributions of this paper are reviews of:

- currently available three-dimensional interaction devices
- methods of gesture design and recognition
- applications of usage
- state-of-the-art in interaction design
- evaluation methods for these designs

Focus is on three-dimensional interaction devices and their application in NUI based HCI. Section 2 gives an overview of current HCI design and introduces related technical vocabulary. There exists a large number of application domains, which is discussed in the following Section 3. NUI systems strongly depend on utilised sensors, considered gesture language and underlying methods to recognise these. An overview of proposed sensors and methods is given in Section 4. As the Leap Motion Controler LMC is a promising device for natural interaction design Section 4.1 focuses on systems based on this device. Evaluation of different interaction systems is important to analyse and compare their efficiency, thus evaluation techniques are reviewed in Section 5.

Throughout this paper, a large number of acronyms is used. Therefore, special reference is made to the acronyms listing at the end of this work.

## 2. Human-Computer Interaction

**Figure 1:**
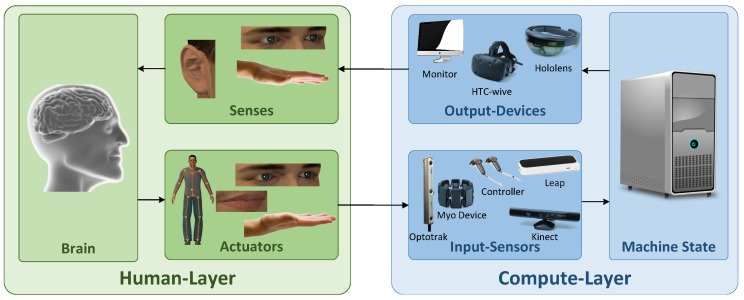
The Human-Computer-Interaction loop.

The term HCI describes the dialogue between human and computer, i.e., the communication channels between humans and computers. At a more general level, the term HCI is replaced by the term Human-Machine Interaction (HMI). The term machine includes computer systems as well as mobile devices or general actuators (e.g., robot end-effectors) the users interact with. The present publication refers to the terms HMI and HCI interchangeably. It is difficult to give an accurate overall definition of these terms. The ACM SIGCHI Curricula for Human-Computer Interaction [2] states that “*HCI is a discipline concerned with the design, evaluation and implementation of interactive computing systems for human use and with the study of major phenomena surrounding them*”. Another definition is, that “*HCI is the process of communication between users and computers (or interactive technologies in general)*” [3]. The expression “interactive” as used in interaction design means the creation of interactive technologies, that support people in everyday situations [4]. There exists a plethora of interactive products one is confronted with nowadays. Uses cases range from microwave ovens and vending machines to entertainment systems, security-critical and medical applications and game consoles. *Smart Environments* represent a special case of interactive systems. The human does not interact with a single machine, but with an array of possibly heterogeneous distributed systems. These systems, in turn, may be conceived as one interaction partner. This aspect leads to additional requirements in the development of interactive systems. In consequence, it is not surprising that goals of HCI design are described as “*to develop or improve the safety, utility, effectiveness, efficiency, and usability of systems that include computers*” [5]. Figure 1 shows a simple version of the HCI interaction loop. The human-layer covers task definition and task performance through the human motor or vocal system as input to the computation layer. Output from the computation layer is received through the senses. On the computation layer input is received through certain input sensors. Input is processed by a computer system affecting its current state and a response is created using e.g., monitors, Head-Mounted Display (HMD) or audio output.

### 2.1. User Interfaces

An important part of HCI is the design of User Interfaces (UIs) for interactive computing systems. Usability of such systems is and will become increasingly relevant for the acceptance of HCI (cf. Section 5). Usability means the extent of the effective, efficient and satisfactory use of a system by a user in a specified context of use [6]. The User Interface (UI) is the interface between computer system and user. Most of the early interfaces are Command Line Interfaces (CLIs) and interaction is performed via keyboard input. A NUI is a special form of a UI. NUI is therefore a special form of a HMI in which the computer system tries to analyse the user’s natural behaviour and acts accordingly [7]. Other aspects are the user’s task performance, comfort, and the system performance [8]. Current changes and developments necessitate a new, expanded way of looking at usability. This concept is represented by the term User Experience (UX) [9]. UX extends the concept of usability by emotional and aesthetical factors, e.g., joy of use or an attractive design. The evaluation of user behaviour is often achieved using sensors. NUI design promises an intuitive interaction that is easy to learn. Monitoring of users can be found in recent concepts, namely, eye, head and body tracking [10], recognition and identification of facial expressions [11] and poses [12], gesture-based interaction on multi-touch devices [13] and in particular mobile devices [14], as well as voice-based interaction [15]. Therefore, it is likely to further differentiate the concept of NUI into the characteristic of Vision User Interface (VUI), Touch User Interface (TUI) and Audio User Interface (AUI) [16]. With the use of this terminology the present work’s focus is on VUI and TUI parts of NUI.

**Figure 2:**
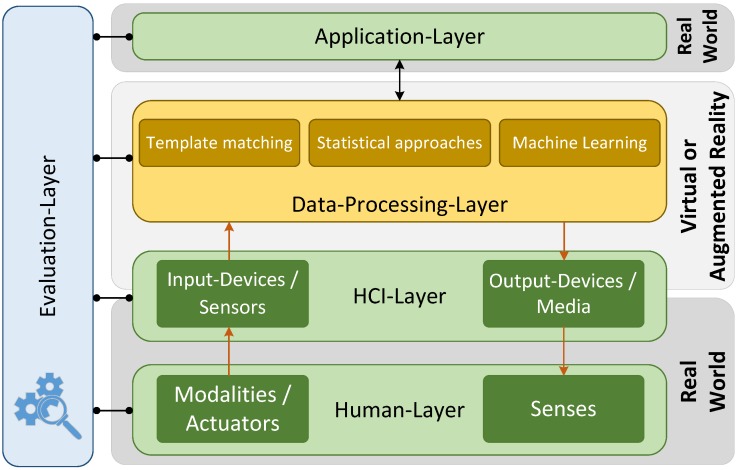
Principles of Human-Computer-Interaction.

In view of those different characteristics of HCI and associated components, Figure 2 tries to summarise the principles of HCI. The figure illustrates the partial components of interaction between human and computer in a wider view. These components differ in the concepts of sensors and actuators (green boxes), communication channels (orange arrows) and data processing algorithms (orange boxes). The data-processing layer covers all aspects of managing user data but also the information related to the environment and covers methods for face or gesture recognition. Data processing algorithms can be separated into three groups [17]: template matching, statistical approaches and machine learning. Template matching means that a prototype (template) of the pattern to detect is available. Similarity measures between the pattern to recognise and the template are represented by statistical models. Approaches are, for example, based on graph matching or graph morphing [18,19]. If the recognition is performed on a set of features, for example, colour, gray level, shape, texture and frequency features or spatio-temporal features [20], the approach belongs to the group of statistical approaches [21,22]. Current works in machine learning mostly refer to the field of deep learning and in particular to recurrent neural networks [23,24]. Thereby, the communication channels will be structured in four specific fields:

- actuators: output signal of the human user
- input-devices/sensors: channel of signals to the HMI-system
- senses: sensory channel of the human user (sensory organs)
- output-devices/media: output signal of the HMI-system

The communication becomes increasingly more complex by the combination of several channels. Further, a distinction is made between multimedia and multimodal interaction. Multimedia interaction describes the concept of using more than one media (e.g., learning system, web-based e-commerce systems) [25]. Multimodal interaction characterises the use of more than one modality to improve system accuracy (e.g., spoken language and detection of lip movement by an optical sensor) [26]. Also associated with this topic are the interaction activities/tasks (action within an application, e.g., selection of an object), interaction technologies (realisations of interaction activities, e.g., selection of an object by using a pointing device) and interaction styles/systems (direct manipulation, voice/language-based, form/dialog-based) [27].

Widespread 2D screen-based interaction systems are Windows/Icons/Menus/Pointers (WIMP)-systems [28], hypertext/hypermedia-systems [29] and contact-based systems [30]. However, this publication focuses on (virtual) 3D-environments. In detail, a further distinction is made between Virtual Reality (VR), Augmented Reality (AR) and Mixed Reality (MR) [31]. VR-systems include displays and tracking systems, but also further application-specific technologies to lead to a perfect immersive impression within the Virtual Environment (VE). AR means a computer-assisted perception or visualisation, which extends (augments) the real world to include virtual objects. Referring to the reality-virtuality continuum [32], MR is defined as a system that can be located anywhere between the extrema of the continuum. The extrema are defined as the real environment (consisting of real objects only) and a fully virtual environment (consisting of virtual objects only). Independent of the precise characteristic of the VE, input- and output-devices are needed [33]. Input-devices can be classified as discrete (e.g., buttons), continuous (e.g., eye-tracker, LMC) and hybrid input-devices (e.g., interactive pen displays). Voice input, as speech input, is mostly used for discrete input tasks, but Harada et al. [34] suggested a *vocal joystick* (non-speech voice input) as a continuous input device. For more details on input devices, please see Section 2.2. Output-devices are, for example, fully immersive displays (e.g., Head-Up Displays (HUDs)), semi-immersive displays (e.g., stereoscopic displays) and haptic or tactile devices. Interaction tasks in virtual environments are navigation, selection and manipulation or system control tasks (e.g., menu-based changes of the system state) [3]. Within the data processing, for example, to recognise human poses or to identify the movement of robots, it is distinguished between low-level and high-level algorithms. Algorithms are often selected considering the applications and contexts (s. Section 3). For Low-Level-Algorithms, refer to the cited literature (Model- [35], Skeletal- [36] Appearance-based [37]) and for High-Level-Algorithms (Statistical model [38]), Machine-Learning [39], Template-based [40]). Focused on contact-free interaction Section 4 discusses gesture detection and recognition methods. Concepts behind specific HCI designs are subject to evaluation (cf. Figure 2). An overview of evaluation methods and their utilisation in the context of three-dimensional interaction is discussed in Section 5.

Since the focus of this work is on interaction, output devices are not considered in detail. The following section gives a short overview of the chronology and evolution of interaction devices in HCI.

### 2.2. Evolution of Interaction Devices

**Figure 3:**
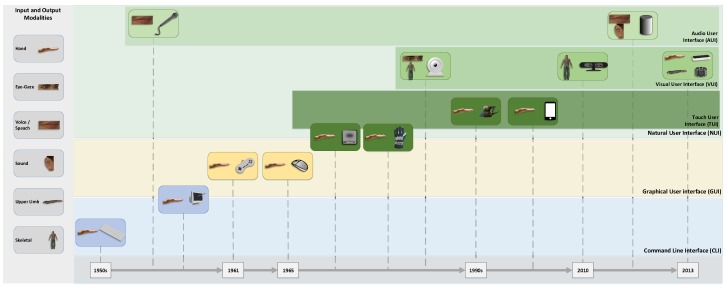
Evolution of Interaction Devices: A time line from keyboard to three-dimensional input devices, classified by the user interfaces these widgets were designed for.

The aforementioned NUI is dependent on sensor devices capturing and detecting the users natural motion to interact with the UI. In the following, a short overview of the evolution from standard input devices to current (natural) interaction devices is presented. Figure 3 gives an overview of this evolution of (natural) interaction design. Early UIs mainly used the **keyboard** as interaction device. These interfaces are referred to as CLI. In 1952, *Bell Labs* developed *Audrey*, the first (fully analogue) speech recogniser used as an voice dialling interface. The first keyboard was introduced with the *Whirlwind Computer* [41] in the same year. As part of the same project, the **light pen** (1955) was invented allowing the interaction with a Cathode Ray Tube (CRT) by affecting the screen with a pen-like object. In 1965, the **mouse** device was developed at the *Stanford Research Laboratory* [42], triggering the development of traditional (two-dimensional) Graphical User Interfaces (GUIs). A mouse cursor pointing at the object to interact with shows to be a more natural metaphor for the selection or activation of an action than keyboard input. With **game controllers** devices for specialised tasks were invented. A first game controller was used in the 1961 *Spacewar!* computer game, consisting of toggle switches in front of the console [43]. In the early 1980s, with upcoming **data gloves**, new and more natural interaction devices were presented, allowing to directly map finger movements to GUI interactions. In the mid-1980s, the *VIDEOPLACE* system [1] introduced the first vision-based interaction system using a **camera** and video projection based approach. In the same period of time Ware et al. [44] proposed an interaction system based on **eye-gaze** detection. Nowadays special devices for eye-gaze detection are available at a consumer level, like the **Tobii EyeX** (Tobii EyeX, https://tobiigaming.com/product/tobii-eyex/ (accessed on 12 March 2018 )) (cf. Section 5.1). Since the early 1990s, **3d mice** (at least three degrees of freedom) became available. At the same time **haptic devices** introduced HCI to users with impaired vision giving haptic feedback from the UI. One widely used haptic input device is the **Phantom Omni** (Phantom Omni, https://www.3dsystems.com/haptics-devices/touch (accessed on 9 March 2018)), which is mainly used in medical applications. Data gloves and haptic devices, besides the vision-based *VIDEOPLACE* input system, could be referred to as the first natural interaction devices. **Touch screen** interaction was introduced in the 1990s with first resistive touch screens. With the invention of **multi-touch** displays and later smart phones, TUI became state of the art. In 2006 a popular representative of advanced 3d mice, the **Wii Remote** (Wii Remote, https://web.archive.org/web/20080212080618/http://wii.nintendo.com/controller.jsp (accessed on 13 March 2018)) was introduced, combining three-dimensional position and orientation detection with motion tracking in gaming contexts. A variant of this controller is the **PlayStation Move** (PlayStation Move, https://www.playstation.com/en-us/explore/accessories/vr-accessories/playstation-move (accessed on 12 March 2018)) controller (2010). As far as VR or MR applications are concerned, specialised controllers with comparable properties like the **Oculus Touch** (Oculus Touch, https://www.oculus.com/accessories/ (accessed on 12 March 2018)) (2016) or the **HTC Vive Controllers** (HTC Vive Controller, https://www.vive.com/eu/accessory/controller/ (accessed on 12 March 2018)) (2016) were invented.

The **Kinect** device (2010) was the first consumer grade depth sensor allowing the tracking of users full-body movements with high precision [45]. Shipped Software Development Kit (SDK) provide fully articulated 20 point body tracking. The Kinect 1.0 device consists of a RGB camera, depth sensor and multi-array microphone. Depth sensor is based on an Infra-Red (IR) laser projector and a monochrome Complementary Metal–Oxide–Semiconductor (CMOS) sensor. Depth images are generated based on structured light, i.e., projecting a known pattern on to the scene and deducing the depth of objects from the deformation of these patterns in the captured images. In 2013, Kinect version 2.0 was released using time-of-flight based range sensing in full-HD resolution, i.e., depth of a pixel in the scene is deduced by measuring the time light travels to a scene object and back. Sarbolandi et al. [46] gives an in-depth comparison of both versions of the Kinect device. The recent introduction of devices like the LMC in 2013 (s. Section 4.1) or the **Myo** Gesture Control Armband (Myo Gesture Control Armband, https://www.myo.com/ (accessed on 8 March 2018)) (2014) device offered natural ways of finger and hand gestures supported interaction on a consumer level. The Myo Gesture Control Armband is a hand and arm gesture recognition device worn on the user’s wrist. It is based on ElectroMyoGram (EMG) sensors for the detection of gestures based on forearm muscle contractions combined with gyroscope, accelerometer and a magnetometer (Inertial Measurement Unit (IMU)) for position, orientation and motion tracking. The EMG unit captures signals with a frequency of 200 Hz and the IMU reports with a frequency of 50 Hz. Recognition of eight hand gestures is provided by the Myo-SDK (Myo SDK Manual, https://developer.thalmic.com/docs/api_reference/platform/index.html (accessed on 13 March 2018)).

With the introduction of *Siri* [47] in 2010, AUIs gained increasing attention on the consumer market. Based on this technology so-called **digital assistants** were created combining speech recognition and synthesis. Interaction based on speech input and response nicely integrates into mobile NUIs for applications like home automation and the Internet of Things (IoT).

IR-based **motion capture** devices like Optotrak (Optotrak, https://www.ndigital.com/msci/products/ (accessed on 12 March 2018)) or Vicon (Vicon Motion Systems, https://www.vicon.com (accessed on 12 March 2018)) are used in articles referenced by this work as ground truth for evaluation tasks (cf. Section 5). For the sake of completeness, reference is made to ElectroEncephaloGraphy (EEG)-based Brain-Computer Interface (BCI) systems [48,49], which allow interaction with the user interface based on brain signals. However, the stage of applications based on these interfaces are not yet beyond the proof-of-concept stage.

## 3. Applications and Contexts

Three-dimensional interaction and appropriate devices are utilised in a variety of applications. These applications are categorised into medical applications discussed in Section 3.1, human-robot interaction (Section 3.2), text recognition (Section 3.3), education and analysis (Section 3.4), music (Section 3.5), games and gamification (Section 3.6) and authentication and identification reviewed in Section 3.7. For short information about the sensors used please refer to Section 2.2.

### 3.1. Medical Field

One of the fields where contact-free interaction is of prime importance is the medical domain because it minimises the risk of infections. This category covers applications used for rehabilitation, physiotherapy, surgery, telemedicine and medical visualisation. Healthcare environments are using a gesture-based interaction and visualisation for touch-free NUIs in combination with a Kinect or LMC sensor. This, for example, is used to explore Computed Tomography (CT) volume datasets [50] or 3D orthodontic models [51]. Corresponding concepts and solutions also enable use during surgery [52,53]. A combination of the Myo gesture control armband and the LMC is used by Hettig et al. [54] in order to compare the deviations of conventional interaction techniques for interventional neuroradiology with gesture-based solutions. For special purpose systems such as the OsiriX (OsiriX, OsiriX foundation, Switzerland, http://www.osirix-viewer.com (accessed on 13 August 2017)) medical image viewer, several plugins with gesture control exist [55,56,57]. In particular, the LMC is suitable to evaluate abnormal finger motions of the trigger finger [58,59] or as a diagnostic tool for impaired hand function caused by cervical myelopathy based on a hand grip-and-release test [60]. The field of applications extends to all aspects of rehabilitation. The usability of the LMC for stroke rehabilitation was investigated within a 3D virtual environment, created as a 3D game with the Unity engine (Unity Engine, https://unity3d.com (accessed on 9 March 2018)) [61]. Interactive systems based on the LMC form another promising method for hand rehabilitation, since this approach leads to more fun and commitment [62,63,64,65,66]. This holds particularly in the case of impairments that occur in the early childhood [67]. In the case of rehabilitation, which aims to improve the mobility of the arm, an application used the structured light sensor (cf. Section 2.2) Intel RealSense (Intel RealSense, https://www.intel.de/content/www/de/de/architecture-and-technology/realsense-overview.html (accessed on 9 March 2018)) [68]. Rehabilitation measures during childhood and early adolescence to regain muscle power and movability of the arm system are documented [69,70] as well as to improve all motor skills activities [71]. An important role here is played by the Kinect sensor. An AR-based 3D hologram training is founded by Lee et al. [72] to improve the capacity for coordination of older adults. Brown et al. [73] found a take-home laparoscopic instrument simulator for surgical education, while Cobb et al. [74] present a survey on simulation in neurosurgery in connection with the use of the Kinect or LMC sensor. The LMC is used in conjunction with a haptic device (Phantom Omni) within a surgical simulation [75]. Especially, myographic gesture-control devices, like the Myo-band, will now also be important, for example, as an interaction solution for upper limb amputees [76] or as an interaction device in a physiotherapy treatment session [77,78]. Sathiyanarayanan et al. [77] additionally use the device to explain metrics for successful executions of arm exercises to patients in physiotherapy. Visualising EMG data from Myo device during arm movements helps patients in performing exercises correctly at home. Suitability of the device as a monitoring device is rated high, but consistent delays lead to frustration and discomfort in interaction tasks.

### 3.2. Human-Robot Interaction

With respect to the methodical base, approaches for human-robot interaction can be divided into the categories: interactive control of a robot and programming robot movement. Due to the precision of novel contact-free sensors (e.g., LMC, Kinect), touch-less control of a robotic arm or in general an automatic actuator is the important part in increasingly complex industrial processes and automation systems, in particular in case of a direct human-robot cooperation or collaboration. The majority of authors introduce a gesture-based user interface for real-time robotic arm control (e.g., universal robot (Universal Robots, https://www.universal-robots.com (accessed on 9 March 2018)), KUKA robot (KUKA, https://www.kuka.com (accessed on 9 March 2018))) [79,80,81,82,83]. It is also demonstrated that gesture-based user interfaces are suitable for remote control of robots (teleoperation) [82,84], also in combination with a HUD [85]. *Kruusamae and colleague* [84] teleoperate a *Yaskawa Motoman SIA5D* to thread needles of different sizes. Findings are that a designed gesture control interface based on LMC is as suitable as a standard interface using a computer mouse. Optical tracking systems are utilised as alternative interaction devices [86]. In addition to use contact-free sensors for robot control, human-robot interaction is considered. Hernoux et al. [87] introduce a system consisting of a LMC and a *UR10* robot (six-axis robot from *universal robots* with a three-finger adaptive gripper). The user’s hand motions and gestures are captured by LMC, interpolated to compensate for sampling limitations and reproduced by the robot. Andersson et al. [88] designed an AR enabled robot simulator based on Robot Operating System (ROS) (ROS, Open Source Robotics Foundation, http://www.ros.org (accessed on 13 August 2017)) and Tsarouchi et al. [89] define a vocabulary of gestures for robot control and integrate it into ROS. Manawadu et al. [90] use a haptic device and a LMC in a VR driver-vehicle interface (DVI) showing that immediate haptic feedback supports lower input times and reduces input errors (cf. Section 5.3). Besides industrial applications, contact-free interaction concepts have been proposed for NUIs to control drones [91,92]. Suárez Fernández et al. [93] present a multi-modal drone navigation system using speech, hand gestures, body position or visual markers. Hand gesture interaction is reported the most natural and fun method for drone navigation in the presented flight-tests in controlled indoor environments. Peshkova et al. [94] give an comprehensive overview of natural interaction techniques for Unmanned Aerial Vehicles (UAVs). Input vocabularies are classified according to their underlying mental models and clustered into three classes (*imitative*, *instrumented* and *intelligent*). In terms of intuitiveness of input vocabularies, single metaphor based gestures and gestures based on multiple metaphors for UAV are presented [95]. The proposed gestures are evaluated (using a Kinect sensor) in a user study. Findings are that gestures based on a one metaphor are rated as more intuitive. In terms of usability and user experience (cf. Section 5.1 and Section 5.2), an interview based user study in gesture based UAV control is presented [96] defining a *coherence score* to evaluate input vocabularies. Variation of requirements for gesture based interaction for drone control are presented by Monajjemi et al. [97,98]. An arm-waving gesture is used to attract a drone from a distance. Afterwards appearance-based tracking is adopted to approach the human until face detection is possible. In close-range an optical flow based gesture detector is used to recognise an arm gesture for interaction with the UAV.

### 3.3. Text Recognition

Several publications present work related to text recognition. These publications can roughly be divided into approaches to recognise general mid-air finger writing and Sign Language Recognition (SLR). Handwritings in mid-air are captured by a contact-free sensor. Recognition methodologies are using the captured hand or arm position trajectories as features and Neuronal Networks (NNs) or Hidden Markov Models (HMMs) (see Section 4) as classifiers to extract human-readable text. Another approach uses video-based hand tracking to recognise characters in one-stroke finger gestures [99]. Chang et al. [100] proposed a framework for mid-air handwriting recognition which is intended for use with wearable egocentric cameras. Furthermore, there are recognition approaches for Chinese [101] and oriental [102] characters.

Most of the studies examined the sustainability of the LMC. They usually provide combinations of heuristics to extract words and machine learning approaches to classify phrases [103,104,105,106]. Publications in the SLR context aim at removing language barriers between deaf or speech impaired people and non-sign language users. Resulting systems are also known as Sign Language to Text Converters (SLTCs). Sign language gestures are captured using a 3D sensor (e.g., Kinect, LMC) and gestures are translated into text. General SLTC-applications are presented, for example, by Fok et al. [107] or Kumar et al. [108]. Country-specific recognition approaches are available amongst others for the American [109], Australian [110], Arabic [111], Greek [112], Indian [113] and Mexican [114] sign language. An evaluation of different potential solutions for recognition, translation and representation of sign language for e-learning platforms has been conducted by Martins et al. [115]. Special mention should also be made to a self-made wearable ring device for text input (TypingRing) [116]. This device allows capturing of text written on any available surface.

### 3.4. Education and Analysis

An intuitive user interface is an important factor for virtual education platforms. Especially the LMC and the Kinect are shown to be a solid basis for interface designs. A general testing environment for interactive educational methods using the Kinect sensor is presented by Moriarty et al. [117]. Adjustment to the user experience is recognisable using artificial intelligence. A virtual reality system, showing the Elmwood Park Zoo, provides new insights into wild animals [118]. Häfner et al. [119] describe a CAVE installation including an ART (ART—Advanced Realtime Tracking, https://ar-tracking.com (accessed on 9 March 2018)) tracking system and a flystick (ART Flystick, https://ar-tracking.com/products/interaction/ (accessed on 9 March 2018)) for interaction. The system is intended for graduate and undergraduate students to improve their engineering education and to analyse practical engineering problems in a (virtual) team. Further, a VR learning platform is also availed for medical education. A virtual anatomical 3D human model is explored interactively with an LMC-based interface [120]. User satisfaction is measured by a questionnaire. Results are that the system can effectively support the 3D anatomy learning process. The zSpace platform is an evidence of the growing potential of the combination of graphical interfaces, VR and modern sensors [121]. Besides the LMC an Emotiv EEG headset (Emotiv, https://www.emotiv.com (accessed on 13 March 2018)) allows to depict complex processes of human interaction. The user can explore space system models, for example, NASA or ESA missions. A similar approach is explained by Salvadori et al. [122]. Computer generated representations of molecular systems are visualised in a CAVE virtual reality system that allows multiple users to interact with molecular sciences and to analyse molecular structures. A list of publications also focuses on the analysis of datasets and the integration in virtual environments, which includes interface technologies like the Oculus Rift (Oculus Rift, https://www.oculus.com (accessed on 9 March 2018)) with LMC to, for example, visualise large geoscientific datasets [123]. Samuel et al. [124] are investigating approaches to explore time-evolving graph data. Performance of activities on a digital scrum board using LMC is described by Rittitum et al. [125].

### 3.5. Music

An interesting field of application for contact-free devices is the transfer from gestures to music. This can be considered in particular as a special form of education (cf. Section 3.4). The LMC is discussed as gesture interface for creating new Digital Musical Instruments (DMIs) [126] and tested interacting with a virtual one octave keyboard. Major problems addressed are tracking loss due to interactions with fingers close to each other, occlusion problems that forced the user to choose an unusual hand posture and varying latency. Using tactile feedback (glass table) resulted in higher comfort and precision. Howell et al. [127] build a Virtual Musical Instrument (VMI) that consists of an Oculus Rift headset and a LMC. The designed interface allows building and playing of music compositions in an immersive environment. One gesture allows users to compose music by arranging notes in a virtual environment and a second gesture allows to play compositions. Besides the creation of music, contact-free devices improve learning platforms. A research project discusses the effectiveness of teaching and performing in a music class with the LMC at an elementary school [128]. Findings are that motion recognition is too inefficient for daily use. Tasks with the LMC are rated too demanding. Further, tracking loss is considered another major issue. Performing and learning musical gestures is also proposed using Kinect and LMC. Hemery et al. [129] present a concept and a prototypical realisation of a NUI supporting the recognition of pianistic gestures. The gestures are transformed into sounds. Volioti et al. [130] designed a platform for learning motor skills of musical gestures. A LMC-based system to interact with particles visualising orchestral arrangements is presented by Fonteles et al. [131] to visualise musical compositions for an audience and the conductor.

### 3.6. Games and Gamification

This section summarises approaches of gamification and gesture-based, sensor-assisted interaction interfaces for games. Gamification describes the use of game design elements in non-game environments [132]. Piker et al. [133] explain the use of the LMC as a gesture-based interaction device for video games. In the same context, a LMC-based dialogue is also used to adapt games for enhancing the fine motor skills of children with autism [134]. In general, LMC-based solutions appear promising interfaces for children. A typical example is the honey bee dance serious game for kids [135]. Simple hand-gestures allow the children to control and thereby learn the bees dance. Another study is dedicated to the development of 3D games for children with motor disabilities [136]. Chastine et al. [137] compared a First Person Shooter (FPS) game implementation using the LMC as user input with traditional gamepad or mouse and keyboard input. Mini-games were designed for movement (obstacle avoidance) tasks, aiming tasks and combinations of both. The study shows that interaction with the LMC is promising only in simple obstacle avoidance tasks (and only compared to gamepad input).

Classical children’s toys are increasingly digitised and transferred to VR applications. In combination with the LMC and the Kinect sensor, an interactive VR allows to simulate real LEGO (The LEGO Group, LEGO System A/S Denmark, www.lego.com (accessed on 13 August 2017)) toys [138], enabling the user to design and manufacture buildings. NUIs for VR games with a LMC gesture-based interaction employ the Unity game engine which is due to the availability of Unity Core Assets and Modules for gesture interfaces with the LMC (Unity Assets for Leap Motion Orion, https://developer.leapmotion.com/unity (accessed on 13 March 2018)). A notable example includes a laptop, a smartphone, and the Freefly VR headset [139]. This proposal for VR games includes virtual panels with hand gestures. Another NUI is intended for use in various board games (e.g., chess) [140]. The study uses the LMC and a lenticular display (3D display) for immersion. Soares et al. [141] provide a variation of the *hangman game*, using the LMC, for learning the alphabet of the Portuguese sign language.

### 3.7. Authentication and Identification

Sensors like the Kinect and the LMC allow contact-free hand recognition. Besides the typical applications in user interfaces, derived geometry features from the sensor can be used for personal authentication and identification. The first approaches used classical Charge-Coupled Device (CCD) cameras for computing hand geometry measurements for a person-related hand recognition [142]. To improve measurements for the recognition of individual hand types, the idea came up to use IR images. In 2012, Guo et al. [143] presented a self-made IR device. The proposed approach describes the computation of hand geometrical features based on the IR images for personal identification. As soon as favourable commercial IR sensors become available, systems for personal authentication using hand-feature detection increased. The inexpensive Kinect sensor is designed to provide depth and colour images (s. Section 2.2). Wang et al. [144] present a hand biometric authentication method based on this device. In addition, also facial (Kinect) images are supported for identification [145]. Texture and shape features are used for personal identification, but also to classify gender and ethnicity. The majority of the publications that examined the impact of the LMC for authentication and identification are from the year 2015. Authentication systems are based on the classification of touch-less mid-air gestures [146], on biometric hand features [147] or on a combination of hand geometry and gestures [148]. Moreover, individual features of the shape and speed of hand movement trajectories and the inclination of fingers are used for identification [149]. Further, a study is evaluating the variability of LMC-based in-air signatures during a period of four months [150]. The results reveal that the LMC could, under certain circumstances, be used for biometric identification as some of the proposed biometric measures show not “too high” variability. The authors explain that further studies are needed to evaluate the abilities of a system with the identified low variability measures to identify the original author of a signature.

As stated by Wigdor et al. [151], all computer vision-based approaches to create NUIs have to be developed towards the goals of robust, real-time and application independent visual tracking, modelling and recognition of humans and their activities. One of the prime concerns is robustness. The usability of NUIs has to be guaranteed not only for laboratory setups, but for arbitrary environments. Thus, the next section gives an overview of proposed methods for reaching these goals.

## 4. Methods

This section gives an overview of proposed methods for the design and recognition of gestures in contact-free 3D human-computer interaction. The purpose of three-dimensional user interaction is to build a model of the communication between human and environment (cf. Section 2). This work focuses on the use of sensor-based devices for interaction design, thus methods for definition and recognition of gestures are addressed. Gestures are naturally classified as either static or dynamic. If the user adopts a certain pose, it is called a static gesture. Dynamic gestures are defined as tracked motions over time. Applications like SLR (cf. Section 3.3) use a mixture of static and dynamic gestures. Thus, gesture recognition is always based on temporal, positional splitting of detected motions or both. In the context of HCI systems, gestures are usually further classified as *hand and arm gestures*, *head and face gestures* and *body gestures* [22]. This work focuses on hand and arm gestures (cf. Section 1), the underlying methods for sensor or vision-based human-computer interaction applications are discussed based on the aforementioned sensors. Sensor-specific feature extraction techniques, as well as gesture recognition algorithms, are surveyed.

Recent work on vision-based approaches to hand detection and hand gesture recognition for SLR are surveyed by Kumar et al. [152]. Reviewed systems are based on skin colour modelling, shape detection, segmentation and contour modelling for hand segmentation and k-Nearest Neighbors (k-NNs), HMMs and Support Vector Machines (SVMs) for gesture detection. Recently, contour-based view independent hand posture descriptors for extraction using a novel signature function and gesture detection by spatio-temporal Hough forests are proposed using a wearable egocentric camera to detect in-air writings [100]. Fingertip detection accuracy of 97.7% are reached and 90.4% in character recognition. Mei et al. [153] propose the combination of a ‘slowest error growth’ discriminant based selection of classifiers to create multi-class classifiers for hand detection describing hit rates of 90.2% with a false positive rate of 0.2% and Zhou et al. [154] utilise a weighted radial projection algorithm using detected wrist position as the origin with gesture recognition rates between approx. 50% to approx. 99%. Rautaray et al. [155] give a comprehensive overview of vision-based hand gesture recognition for human-computer interaction, reviewing over 250 papers. Appearance-based hand gesture representations are found preferred over 3D based representations. Most reviewed applications are desktop applications. Limiting factors of the presented recognition systems are poor image resolution, low frame rates, varying illumination conditions and occlusion problems. Static and dynamic sets of backgrounds from which hands are segmented are concerned as a major problem.

With the broad availability of depth-based sensors, the design of natural contact-free user interfaces gained increasing attention. Suarez et al. [156] gives a comprehensive review of 37 papers discussing depth-based hand tracking and gesture recognition. The separation of gesture recognition systems into three components is supposed. First, depth-data is acquired depending on the underlying depth sensor. After data acquisition, the object to track (hand) is localised in the depth-data and finally, tracked object-data or accompanying trajectories are classified as a certain pose or gesture. As this split seems natural it is furthermore suitable to model general gesture recognition systems for all the aforementioned sensor frameworks and thus builds the basis for the following elaborations. Findings are, that hand localisation and tracking is achieved using Kalman filters and mean shift. Classification is based on HMM, k-NN, Artificial Neural Network (ANN), SVM and finite state machines. Approaches for challenging environments with low lighting or occlusions are missing.

Kumar et al. [152] discuss a multimodal framework using the LMC and a Kinect sensor for SLR based on HMM and Bidirectional Long Short-Term Memory (BLSTM)-NN classifiers plus the combination of both. Findings are, that gesture recognition accuracy is improved by fusion of both sensors. If classifiers are combined, overall accuracies of 97.9% for single-handed gestures and 94.6% for gestures involving both hands are achieved. The HMM-based classifier shows 97.4% and 93.6% accuracy and BLSTM-NN-based classifier 87.6% and 83.4%.

Regarding authentication methods using the Kinect sensor (cf. Section 3.7), Wang et al. [144] show finger length calculation results based on geodesic contour analysis of over 92.7% and identification rates for finger length of more than 79.4%, hand curvature of more than 80.4% and more than 81.5% for the combination of both measurements. Resulting authentication accuracies of more than 80% are reported for a combination of geometrical and RGB based measurements.

EMG based hand gesture recognition literature follows an analogous paradigm. Data acquisition is followed by a feature extraction phase based on common statistical features [76] or Fourier-based features like Fourier variance, fundamental frequency or spectrum length [157] or extracting features based on sliding window techniques [158]. Matos et al. [76] identified leg gestures with the Myo sensor (see Section 2.2) by division of gestures into five moments and a ranking approach to provide a HCI solution for upper limb amputees. Boyali et al. [159] use the Myo sensor to detect the eight Myo hand gestures (s. Section 2.2). Spectral Collaborative Representation based Classification (CRC) using the Eigenvalues of observed signals as features is used for gesture recognition with an accuracy of more than 97.3%. Kim et al. [157] used a single channel EMG sensor attached on the forearm to identify hand gestures. Real-time classification is achieved using a decision-tree based combination of two linear classifiers, k-NN and Bayes, with an accuracy of 94% using a combination of common statistical and spectral features. Georgi et al. [158] fused IMU worn on the wrist and a 16 channel EMG sensor attached to the forearm to infer finger and hand movements. Twelve gestures were identified and recognised using HMMs as classifiers. Recognition accuracies are 97.8% for session-independent and 74.3% for person-independent recognition. Hasan et al. [160] use EMG signals captured from dominant forearms of the subject to recognise *check*, *dislike*, *good luck* and *hang loose* gestures using feed forward ANN for gesture recognition.

Upon the design of new feature extraction or gesture recognition methodologies, publicly available datasets for testing purposes become increasingly meaningful. Cheng et al. [161] present a survey on three-dimensional hand gesture recognition including listings of data sets for static, trajectory and American sign language gestures.

The following section reviews gesture definition and recognition methods of LMC-based systems proposed in current literature.

### 4.1. Leap Motion Controller

**Figure 4:**
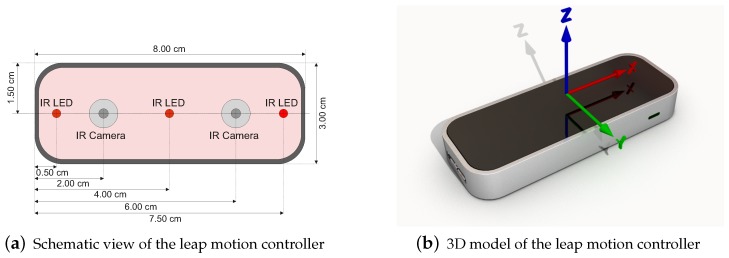
Visualisation of a (**a**) schematic view [162] and (**b**) 3D model of the leap motion controller with corresponding right-hand coordinate system [169].

The Leap Motion controller is a consumer-grade contact-free, marker-less sensor developed by *Leap Motion* (Leap Motion, https://www.leapmotion.com/ (accessed on 13 August 2017)). It is primarily designed for hand gesture and finger position detection in interactive software applications. Tracking of arm, wrist and hand positions of up to four individuals and the tracking of stick-like tools are supported. Figure 4a shows a schematic view of the controllers hardware setup. Besides three IR emitters, the device incorporates two CCD cameras. Despite popular misconceptions, the LMC does not create a depth map of the scene or emits some sort of structured light. In order to get objects’ positions from the stereo-vision images, all the calculations are performed on the host computer using a proprietary algorithm. As stated by the manufacturer, sensors accuracy in fingertip position detection is about 0.01 mm. Robustness and accuracy of the LMC in different areas of application have been discussed in detail [162,163,164,165,166,167] (cf. Section 5.5). Fingertip positions over LMC are measured in Cartesian coordinates relative to the centre of LMC in a right-handed coordinate system (cf. Figure 4b). The hand tracking speed is up to 200 frames per second in a 150° field of view with approximately eight cubic feet of interactive 3D space [168].

**Figure 5:**
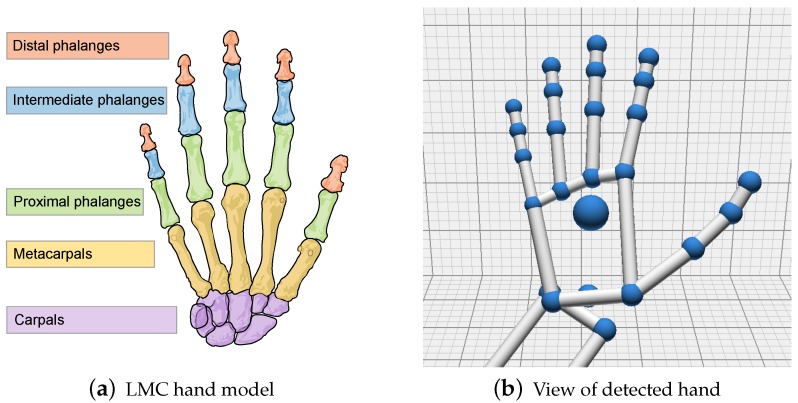
The LMC hand model provides access to positions of single bones in the tracked hand: metacarpal, proximal phalanx, intermediate phalanx and distal phalanx are tracked for each finger (thumb modelled with 0-length metacarpal): (**a**) hand model (Original diagram by *Marianna Villareal* https://commons.wikimedia.org/wiki/File:Scheme_human_hand_bones-en.svg (accessed on 13 August 2017)) used by LMC-SDK; (**b**) view of detected hand.

Besides fingertip position and hand gesture detection, the LMC-Application Programming Interface (API) (Leap Motion SDK and Plugin Documentation, https://developer.leapmotion.com/documentation/index.html?proglang=current (accessed on 15 August 2017)) provides access to a skeleton model of the tracked hand. Positions of single bones including metacarpal, proximal, phalanx, intermediate phalanx and distal phalanx are modelled (cf. Figure 5). Queries such as positions of each finger(-bone) in three-dimensional space, open hand rotation, grabbing, pinch strength, etc., are supported. For detailed description of accessible arm, hand and finger features please refer to the API documentation. Access to raw device data is provided by recent versions of the API. Raw data of the two captured images contains the measured IR brightness values and the calibration data required to correct for complex lens distortion. VR-based publications [69,105,118,121,123,127,139,170,171,172,173,174] mainly use motion tracking capabilities of the LMC to map the user’s hand to a virtual hand. There also exists a wide range of publications discussing the use of LMC to directly map non-VR application controls (e.g., camera translations and rotations) to hand movements (e.g., palm or fingertip movement and hand rotation) [53,55,56,93,120,124,126,128,133,137,168,175,176,177,178,179,180,181,182,183,184,185,186,187,188,189,190].

In the previous section *sensor data acquisition*, *object detection* and *object tracking* were identified as the three components of gesture detection systems. As this work’s special focus is on the LMC and accompanying methodologies, essential components of LMC based interaction systems are defined differently. Hand and finger detection is performed by the LMC such that object detection is not in the focus of related works. Gesture detection based directly on the IR images of the LMC is presented by Mantecón et al. [191]. IR images from the device are used to compute a *Depth Spatiograms of Quantized Patterns* based feature vector and *Compressive Sensing* for dimensionality reduction. Resulting features are classified using SVMs. Recognition accuracy observed is greater than 99% for a set of 10 static gestures. Most of the current literature discusses the incorporation of the LMC into existing user interfaces or using it in combination with other sensors. The transformation from LMC coordinate system to the applications or other interfaces coordinate system is a relevant task. Thus, three essential components of LMC-based interaction systems are defined as:

- Data acquisition—transformation between LMC coordinate system and the coordinate system of the scene of interaction, data fusion and smoothing.
- Feature extraction—obtain features of tracked hand(s) or arm(s).
- Gesture definition and recognition—define and recognise gestures to be detected by LMC.

In the following subsections, proposed methods according to these research questions are presented.

#### 4.1.1. Data Acquisition

Finger and hand position data from the LMC is recorded in coordinates regarding the centre of LMC (cf. Figure 4b). To embed LMC-based interaction in existing systems, coordinate transformation from LMC coordinates to system coordinates is required. This is accomplished by affine transformation [192]. In case of sensor data fusion, i.e., the transformation of different position measurements with different sampling rates to one system, techniques like Kalman filter [193] or other particle-based methods are utilised. Silva et al. [194] presented the use of sensor data fusion techniques based on Kalman filter to combine data from LMC and Myo sensors in order to create a 3D virtual simulation of an arm motion. For compensation of possible tracking errors of the LMC Kalman filtering is also used to optimise the estimation of hand position and orientation [101,195]. Marin et al. [196] jointly calibrate the LMC and a depth sensor by mapping detected fingertips of the open hand from LMC space to depth sensor space and Kumar et al. [152] use a linear interpolation based resampling technique to combine both sensors for SLR.

#### 4.1.2. Gesture Definition

The LMC-API is shipped with built-in recognition capabilities for four different gestures. The so-called *circle gesture* is triggered by a one finger circular movement, the *swipe gesture* is activated by straight line movement of the hand with extended fingers and the *screen* and *key tab gesture*, which are triggered by forward and backward or upward and downward tapping movement of a finger. Besides built-in gestures, only a few differing gestures are proposed. The *pointing at* gesture is proposed, where the selection is either triggered by a move in the pointed direction [85] or by a *wait to click* [173] mechanism. Further simple hand movements are considered as discrete gestures, like a *push gesture* towards or away from the LMC [118,197], a *clap gesture* or a *two-handed swipe* [118]. Further simple *position thresholding* [54,126] is also used to perform discrete inputs. Interaction with scene objects is realised by *grabbing* or *grasping* [113,198,199,200]. Here selection is recognised by collision detection between the object of interaction (e.g., a virtual hand model) and the scene object. *Vivian* [201] presents a grammar of mid-air gestures for the LMC. Six atomic gestures recognised by the LMC are defined which build the basis for an extended Backus-Naur for describing the language of interaction with the LMC. Zaiti et al. [202] performed an elicitation experiment collecting gestures for free-hand television (TV) control and gave implications for the design of hand and finger gestures for TV control. Findings were that user-dependent gesture training is recommended and previously acquired gesture interaction models should be exploited. Two-dimensional gestures were preferred and users łproposed to draw letters in the air to execute commands starting with these letters. Rempel et al. [203] present a measure for the comfort of different sign language gestures as performed over the LMC. The most comfortable position of the hand during interaction was at lower chest height and close to the body. Smooth movements at this height were considered comfortable, while movements at shoulder or face height were considered least comfortable. Hand postures with straight wrist and palms facing each other were considered comfortable. Extended or abducted fingers, fingers in a *claw* shape or in a tight fist were considered least comfortable.

Robot (tele-)operation user interfaces (cf. Section 3.2) benefit from three-dimensional interaction devices. Positions of the users hand or gestures are mapped to robot motions [81,204,205] or the position of the robots end-effector [84]. Here inverse kinematics have to be taken into account. Li et al. [206] used the LMC to build a kinematic calibration scheme for an end-effector with extended workspace by bending its deformable links according to different tasks. The plane-of-rotation is used to obtain the initial kinematic parameters by Random Sample Consensus (RANSAC) and Least-squares algorithms.

#### 4.1.3. Feature Extraction and Gesture Recognition

The LMC-API allows access to a rich features set for detected hands or arms. Twenty-one features are composed to so-called frames and made available through the API. Only parts of these features are utilised in the majority of proposed methods for gesture detection in current literature.

If the LMC is used for the measurement of tool positions, accuracy or performance is evaluated, fingertip (or tool) positions are simply tracked over time [65,162,163,164,165,166,167,169,207].

Biometrical hand recognition based authentication systems (cf. Section 3.7) measure the length of the carpals and distances from fingertip to palm. Chahar et al. additionally capture behavioural information from sequences of LMC gestures and times between gesture activation [146] using a Levenshtein based algorithm measuring the similarity of two gesture sequences. Classification is based on Naïve Bayes (NB), ANN, Random Decision Forest (RDF) classifiers, exploring match-score level fusion to analyse the effect of combining scores obtained from different classifiers, achieving 81% genuine accept rate at 1% false accept rate. Chan et al. [148] used all 104 attributes of hands and arms captured by LMC followed by three attributes of the circle gesture in order to recognise a static login gesture and 137 attributes to authenticate a user while interacting with the system using LMC gestures accessible through the API. Classification by Random Forest Classifier (RDFC) with more than 99% accuracy in static mode and more than 98% accuracy in dynamic authentication show high potential of the proposed system. Position, speed and the inclination of fingers are used to identify users by Kamaishi et al. [149]. Aslan et al. [208] found that recognising five fingers, track fingers over a period of time, and normalising mid-air gesture data were the main issues for the design of authentication gestures with concern for reliable recognition. Normalisation of the data based on the centre of the palm, tip of the index finger and the pinky finger was used. Discrete Time Warping (DTW) was used to identify gestures from normalised data series, observing accuracy rates between 86% and 91% for two different kinds of mid-air gestures in different contexts.

VR applications use the LMC to map a virtual hand to the users hand by using the skeletal model provided by LMC-API and different visualisation and interaction frameworks to create interactive scenes with robust collision detection [118,120]. Also, desktop applications use the LMC to interact with three-dimensional synthetic objects. *Stoyan* [209], for example, used the LMC to map object manipulation tasks to the users hand movements.

In the context of SLR, a long list of classifiers are used. It should be mentioned that the different approaches for the recognition of sign language are based on different gesture sets consisting of gestures with varying degrees of difficulty. Thus, a direct comparison of the results is not appropriate. Chuan et al. [210] use k-NN and SVM based recognition strategies with recognition accuracies of 72.8% and 79.8%. Geometric Template Matching (GTM), ANN and Cross-Correlation (CC) are used by Khan et al. [109] to create an SLR to text converter with accuracies of 52.6%, 44.8% and 35.9%. Using the difference between palm and the phalanges as features and ANN for recognition accuracy of 99.3% are reached, while using the length of the phalanges reaches 99%. Using two Leap Motion Controlers (LMCs) in perpendicular arrangement and fuse both outputs by covariance intersection and Kalman filter using fingertip, joints and palm position as input features, gesture recognition with an accuracy of more than 84.7% is observed. Kumar et al. [152] use a multi-modal approach combining the Kinect sensor with an LMC. Gesture recognition is performed by HMM or BLSTM-NN. Single-handed gestures are recognised with an overall accuracy of 97.9% using data fusion of both sensors and the combination of both classifiers (97.4% HMM, 87.6% BLSTM-NN). Double handed gestures are recognised with an accuracy of 94.6% (93.6.8% HMM, 89.5% BLSTM-NN).

In the field of application of rehabilitation (see Section 3.1) Gieser et al. [67] use decision trees, k-NN and SVM for static gesture recognition with resubstitution errors of ≤23%, ≤0.5% and ≤2.1%. Vamsikrishna et al. [211] show that Linear Discriminant Analysis (LDA), SVM and HMM as well as combinations of these classifiers perform with average accuracies of 87.7% (LDA), 88.4% (SVM). Combinations of the classifiers perform at the same high level of 98.7% (SVM + Condidtional Random Field (CRF)), 99.4% (LDA + CRF), 98.6% (SVM + HMM) and 99% (LDA + HMM). Lu et al. [212] use Hidden Conditional Neural Field (HCNF) classifiers on two LMC datasets with accuracies of 95% and 89.5%. Manuri et al. [213] study the effect of different lighting conditions to LMC build in gesture detection. Findings are that dynamic gestures are recognised with an accuracy of 78% at 400 lx and 800 lx and only 2% at ≥1000 lx, static gestures are detected with 90% accuracy ( 400 lx and 800 lx) and 85% at ≥1000 lx.

Avola et al. [175] adopted free-hand drawing methods to define and recognise hand gestures based on the LMC fingertip position data. Trajectories are split into a set of two-dimensional projected strokes and convex hull based geometric measures were extracted. Recognition was performed by describing these symbols in *SketchML* [214] language and matching of descriptions.

Table 1 gives an additional short overview of described and additional methods for feature extraction and gesture recognition using the LMC.

## 5. Evaluation

**Figure 6:**
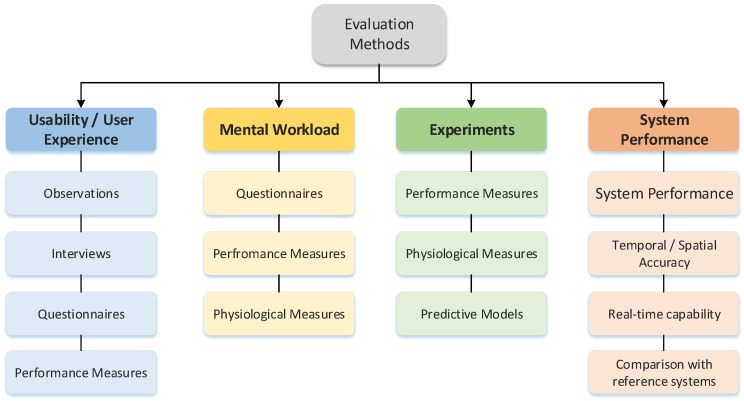
Overview of the evaluation methods in the context of the current review.

The evaluation (cf. Figure 6) of the devices addressed in the context of the current review is mainly related to UI evaluation. Evaluation of UIs is the analysis, assessment, and testing of a UI as a whole or in parts of it [3]. The main purpose of UI evaluation is the identification of usability problems or issues that hinder the user to interact in an optimal way with the UI. Detailed classifications of 3D UI evaluation methods are suggested for example by Bowman et al. [235] and in an upgraded version by LaViola et al. [3]. For example Bowman et al. suggested that usability evaluation methods can be classified according to three key characteristics: involvement of representative users, context of evaluation, and types of results produced. The first characteristic discriminates between those methods that require the participation of representative users and those methods that do not. The second characteristic describes the type of context in which the evaluation takes place, namely those methods that are applied in a generic context and those that are applied in an application-specific context. The third characteristic identifies whether or not an evaluation method produces (primarily) qualitative or quantitative results. The results produced by an evaluation can be distinguished in three different metrics, subjective response metrics, task performance and system performance metrics. In the following, the different evaluation methods found in the reviewed studies are organised according to these metrics and typical studies are to be presented as examples. Subjective response metrics refer to the personal perception and experience of the interface by the user and usually measured via questionnaires or interviews and may be either qualitative (descriptive) or quantitative (numeric). Most of the reviewed studies with users can be subsumed under usability studies in which are the usability or user experience of the device are tested. In general, **usability studies** asses how the characteristics of a device or interaction technique affect the user’s use of the artefact. There are many aspects of usability, including ease of use, learnability, user task performance, user comfort, and system performance [236]. Although one could argue that the classic dimensions of usability already include the users experience, the investigation of **user experience** is considered separately, which covers additional aspects like usefulness, emotional factors and perceived elegance of design [237]. User task performance refers to the quality of performance of specific tasks in a 3D application, such as the time to navigate to a specific location, the accuracy of object placement, or the number of errors a user makes in selecting an object from a set. Investigations of user performance are closely related to **mental workload** measures. Mental workload is thought of a construct reflecting the interaction of mental demands imposed on users by tasks they attend to. Mental workload and can be assessed by task performance measures as well as subjective measures like questionnaires. The evaluation of task performance, mental workload and usability is not only assessed in applied but also in experimental settings. **Experimental studies** are conducted in more generic contexts and usually are focusing more directly on the cause-effect relationships of the different metrics in the context of specific devices. **System performance**, finally, refers to typical computer or graphics system performance, using metrics such as frame rate, latency, network delay, and optical distortion. The evaluation of system performance is crucial because it has an effect on interface performance and user performance. Therefore, we will only address the evaluation of system performance insofar as they affect the user’s experience or task performance. Figure 6 gives an overview of different evaluation methods and examples of their implementations. For information on the devices (sensors) mentioned in the following sections please refer to Section 2.2.

### 5.1. Usability

A widely used measure to assess perceived usability is the System Usability Scale (SUS). This scale developed by *Brooke* [238] is a tool to quickly assign a global scale to that perspective and has been widely adopted over the years [239,240]. There are several contexts were SUS was used [53,133,173,198,241]. For example, the study of Nestrov et al. [53] assessed the perceived usability of LMC and Kinect 2.0 in a clinical context (control of radiological images during surgery). Interestingly, even LMC provided a higher accuracy the usability of the Kinect was higher rated. *Pirker and colleagues* [133] used SUS to explore the LMC as gesture controlled input device for computer games in comparison with traditional keyboard controls. Deng et al. [198] used SUS (in addition to task completion time and error measures) to assess the usability of object manipulation in VR with eye gaze (Tobii EyeX) and hand gesture control (LMC). *Coelho and Verbeek* [241] assessed pointing tasks in 3D VR space and compared LMC and mouse as input devices and used SUS to compare the usability between the two input systems. Caggianese et al. [173] compared two object manipulation methods with the LMC in VR space and used the qualitative data of SUS to compare the perceived usability of the two methods.

Apart from SUS, other studies adopted different usability metrics to investigate 3D interaction methods. For example, Falcao et al. [181] evaluated the interface usability of the LMC for the designer activity while interacting with Photoshop CS6 software. Two metrics were used (performance and satisfaction). The authors concluded that the usability of the LMC is not sufficient yet to replace the traditional mouse and keyboard in the activity of the designers using graphic software. In the paper of *Vosinakis and Koutsabasis* [188] a usability evaluation of four common visual feedback techniques in grasp-and-release tasks using bare hand interaction was conducted. The techniques are ‘object colouring’, ‘connecting line’, ‘shadow’ and ‘object halo’. The usability was examined in terms of task time, accuracy, errors and user satisfaction. A software test bed was developed for two interface configurations: using the LMC alone (desktop configuration) and using the LMC with Oculus Rift (VR configuration). The results show that user performance is better in the VR configuration compared to the desktop. Furthermore, the study showed differences in the usability of the feedback techniques in general and in dependence of the user interface. The study of Barbieri et al. [242] conducted a comparative evaluation of different design alternatives related to the user interaction with virtual museum systems (test bed). The interaction with the virtual systems was either performed with a trackball or with a touchscreen console. For the evaluation traditional metrics and alternative metrics have been used. The virtual museum system should serve a threefold purpose: easy to use, enjoyable, and educative. Traditional metrics have been used to measure performance measures (time and number of errors) to assess aspects related to user satisfaction. As there was less time in testing, the users’ alternative metrics have been employed for enjoyability and knowledge transmission through custom-designed questionnaires and by measuring the exploration times of the two interfaces.

### 5.2. User Experience

As already stated above, usability and user experience cannot be seen as separate but rather complementary measures. User experience is an important supplement of the usability concept. The international standard on ergonomics of human system interaction, ISO 9241-210 [243], defines user experience as “a person’s perceptions and responses that result from the use or anticipated use of a product, system or service”. According to the ISO definition, user experience includes all the users’ emotions, beliefs, preferences, perceptions, physical and psychological responses, behaviours and accomplishments that occur before, during and after the use. Different approaches with varying extent of user experience measures were realised in the reviewed studies. Some of the studies are mentioning and discussing user experience, however rather (if any) employ usability measures. Rather fewer studies are employing validated questionnaires whereas other studies use customised questionnaires. For example in the study of Adhikarla et al. [244] the design of the direct 3D gesture interaction with a light field display was compared with a 2D touch display. The light field display was controlled by the LMC. Besides performance and workload measures (*NASA - TLX*, see Section 5.3), perceived user experience was evaluated for the two interaction conditions by means of a standardised User Experience Questionnaire (UEQ) [245]. This questionnaire allows a quick assessment of the user experience of interactive products and provides six subscales for *Attractiveness*, *Perspicuity*, *Efficiency*, *Dependability*, *Stimulation* and *Novelty*. Recently also a short version of the UEQ was published [246].

Other studies, for example the one from Seo et al. [247], employ rather customised measures for the assessment of user experience. Smart home environments are investigated which require a seamless integration among humans, physical objects and user interactions. In the study of *Seo and colleagues*, a hybrid reality-based approach was employed which allowed the users an egocentric virtual reality and exocentric augmented reality interaction. Immersive visualisation and hand gestures were supported by Oculus Rift DK2 and LMC attached in front of the HMD. In this study user experience measures were adopted from different studies and tailored for the specific research question. Aspects such as *Ease of Use*, *Efficiency*, *Usefulness*, *Stressfulness* and *Immersion* were measured in the custom-designed questionnaire. The disadvantage of using non validated customised scales is, however, that the quality of the measurement may suffer in comparison to validated questionnaires.

### 5.3. Mental Workload

A further fundamental design concept in HCI and Ergonomics (Human Factors) is mental workload (sometimes referred to as Cognitive Load). It is a standard practice to assess mental workload during system design and evaluation in order to avoid operator overload in a variety of HCI contexts. Workload is thought of as a mental construct reflecting the interaction of mental demands imposed on operators by tasks they attend to. The capabilities and effort of the users in the context of specific situations moderate the workload experienced by the operator. Workload is thought to be multidimensional and multifaceted. The principal reason for measuring workload is to quantify the mental cost of performing tasks in order to predict operator and system performance [248]. The measures of mental workload can be categorised in subjective measures, task performance measures and physiological measures [249]. Subjective measures rely on the analysis of the subjective feedback provided by humans interacting with a technical artefact. The feedback usually takes the form of a survey or questionnaire. One of the most known methods are the *NASA Task Load Index (NASA-TLX)* [250] which provides a standardised multi-dimensional scale designed to obtain subjective workload estimates. The procedure is based on a questionnaire and derives an overall workload score on the basis of a weighted average of ratings on the following six subscales: *Mental Demands*, *Physical Demands*, *Temporal Demands*, *Own Performance*, *Effort* and *Frustration*. The questionnaire was used in several studies considered in this review. For example, *Ramirez-Fernandez and colleagues* [66,162] used *NASA-TLX* to determine the mental workload of patients while they performed a motor hand therapy either using a low-cost robotic device (Novint Falcon haptic device) or the LMC as a gesture sensor. In a study of Hettig et al. [54] *NASA-TLX* was employed to assess subjective workload in the comparison of four different interaction methods with radiological image data and volume renderings within a sterile environment. The authors evaluated two gesture input modalities (Myo Gesture Control Armband and LMC) versus two clinically established methods (task delegation and joystick control). In a simulated driving study of Manawadu et al. [90] *NASA-TLX* served to assess subjective workload in the comparison of haptic and gesture driver-vehicle interfaces (joy stick vs LMC) in the context of autonomous driving. *Adhikarla and colleagues* [244] studied free-hand interaction with light field displays by using the LMC and assessed the differences in mental workload between a 2D touch control condition and 3D interaction tasks. In general, subjective workload measures were not used as a single evaluation tool but were usually combined with other measures such as task performance and usability measures.

Performance measures of cognitive load are used far less often in the domain of user interface validation. Performance measures of workload can be classified into two major types: primary task measures and secondary task measures [248]. In most investigations, performance of the primary task will always be of interest as its generalization to in-service performance is central to the study. In secondary task methods, performance of the secondary task measures the load of the operator which is indicated either by the drop of performance either in the primary or in the secondary task [251]. The measures of both tasks are usually objective performance measurements such as the time to complete a task, the reaction time to secondary tasks and the number of errors on the primary task. However, the assessment of mental workload based on secondary task measures seems to be used rather rarely in the context of 3D interaction devices. An example of such an approach was provided by a study of Franz et al. [233] in which the influence of a secondary task on the performance of a primary task was investigated. Users had to perform a primary task with a keyboard and mouse and a secondary task either with the mouse or with 3D gestures performed with the LMC. The results showed that secondary task performed with 3D gestures reduced time on task for primary tasks, which were heavily dependent on the keyboard. For primary tasks, which were more dependent on the mouse, the time results indicate that the use of gestures to perform secondary tasks were less effective. Such findings may help to design secondary tasks in VR environments according to the interference with different resources [251]. It is assumed that mental workload is essentially physiological [3], therefore various techniques have been used, including those measuring heart rate, pupil dilation, and eye movements, and brain activity using EEG or functional Near-InfraRed Spectroscopy (FNIRS). EEG and FNIRS have shown promising results to address mental load, but gathering good data and interpreting results is not easy. Therefore, there is increasing effort to combine such physiological methods with methods of machine learning [252]. A recent neuroergonomic study of *Carrieri and colleagues* [226] used FNIRS measures to objectively evaluate cortical hemodynamic changes occurring in VR environments. There is emerging evidence that FNIRS provides a suitable measure of mental workload [253,254]. The aim of the study of Carrieri et al. was to investigate cortex activity in subjects while performing a demanding VR hand-controlled task. The hand-controlled task was thought to simulate the interaction with a real, remotely-driven, system operating in a critical environment. The hand movements were captured by the LMC. Participants were instructed to guide, with their right hand and forearm, a virtual ball over a virtual route with some critical points. A bilateral ventrolateral prefrontal cortex activation, in response to the hand controlled task execution, was observed in all the participants. These results in general confirmed the feasibility and suitability of FNIRS technology to objectively evaluate cortical hemodynamic changes occurring during the interaction with VR environments.

Other studies in the context of physiological workload measures try to use sensors to measure the workload of the user. There are recent studies which suggest that contact-free camera-based measurement of physiological workload measures are possible. For example, *McDuff and colleagues* [255] measured heart rate, breathing rate and heart rate variability of participants under different cognitive stress levels. Stress was induced by a problem-solving task and a control task. They used the Photo PlethysmoGraphy (PPG) technique to detect volumetric changes in blood in peripheral circulation in the face of participants by means of a simple webcam. Procházka et al. [256] used the Kinect depth sensor to measure breathing and heart rate. Breathing frequency was obtained from the image and IR data of the mouth area and from the thorax movement that was recorded by the depth sensor. Spectral analysis of the time evolution of the mouth area video frames was used for heart rate estimation.

Most of the studies considered in this review do not employ a single evaluation method but combine different methods such as performance measures, workload measures and usability ratings (e.g., [53,242]). Such studies are usually performed in a specific applied context. For example, *Nestorov and colleagues* [53,178] evaluated natural user interface devices for touch-free control of radiological images with the Mouse, LMC and Kinect. In this study, the authors combined user performance tasks with a usability questionnaire. In the performance task users were instructed to achieve a predefined task with each device. The task involved scrolling to a predetermined image, zooming and measuring an anatomical structure. After, the task participant rated the perceived usability of the three devices with the SUS questionnaire [238]. The dependent variable was the speed and accuracy of the task performances for the three as well as the usability scores for the three devices. The study revealed that the LMC was superior and comparable with that of a computer mouse. In general, the study suggests that natural user interface systems for gesture control of radiological images using commercial sensors are feasible to enable touchless interactions in sterile clinical environments. *Ramirez-Fernandez and colleagues* [66] investigated the LMC in the context of a motor rehabilitation task. Two age groups (healthy adults and elders with hand motor problems) used a low-cost haptic device (Novint Falcon) and the LMC. Participants conducted the same rehabilitation task by using a non-immersive virtual environment and the task execution time and accuracy for both devices was measured. As subjective data, mental workload and usability was measured. The study showed clear differences for all participants between the two devices, namely, the precision of the haptic device was better than that of the LMC and participants in the older adult group demonstrated a lower mental workload while using the haptic device.

### 5.4. Experimental Approaches

Besides the applied studies, there are studies which are conducted in a more generic context and usually focus more directly on the different devices, e.g., [169,257,258]. These studies are often based on predictive models [259] to compare the achieved performance of different devices. The most used predictive model is Fitts’s law [260] which predicts how quickly a user will be able to position a pointer over a target area based on the distance to the target and the size of the target. There are different versions of Fitts’s task. For example, Bachman et al. [169] employed the original version of the task suggested by Fitts [260] to compare the achieved performance of the LMC and the computer mouse. Participants had to perform lateral one-dimensional pointing movements between two target areas with the LMC and the mouse. The distance and size of the target areas were manipulated to vary the difficulty of the pointing movements. The results clearly showed that the LMC’s performance in comparison to the mouse was rather limited, which seems to be in contrast to the findings of the study of Nestorov et al. [53] used the LMC in more complex manipulation tasks. However, both evaluation methods provide important information about the possibilities and limits of the LMC. LMC can serve as a useful device in the context of sterile clinical environments, however, its usability as an input device for everyday generic computer pointing tasks is rather limited. Other studies which used Fitts’s Law are based on a two-dimensional pointing task [258], which was suggested by *Soukoreff and McKenzie* [261], or even use 3D versions of the task [241]. A disadvantage in using performance models like Fitts’s Law is that the studies have to be based on a large number of experimental trials on a wide range of generic tasks. Performance models are quite often subject to criticism (e.g., the model doesn’t apply to a particular type of task). Nevertheless, such models provide objective measures of the achieved performance in the interaction with devices and thus can provide important guidance for designers.

### 5.5. System Performance

Measures of system performance are conducted with or without human participants. One of the first studies that evaluated the accuracy and repeatability of the LMC was provided by Weichert et al. [162] and was conducted with an industrial robot. The robot controlled the position of a reference pen with an accuracy of 0.2 mm. Static and dynamic test runs were conducted and the deviation between a desired 3D position and the average measured positions are measured. This difference was below 0.2 mm for static setups and below of 1.2 mm for dynamic setups. A similar study was conducted by Guna et al. [165], which also performed a set of static and dynamic measurements without human participants. For the static measurements, a plastic arm model simulating a human arm was used and several reference locations were selected to assess accuracy across the controller’s sensory space. For the dynamic measurements, two tracking objects with a constant distance were used for two human fingers. For the static measures, a similar high accuracy was reported by Weichert et al. The dynamic measures revealed an inconsistent performance of the controller, with a significant drop in accuracy for samples taken more than 250 mm above the controller’s surface.

Studies of system performance with human participants usually compare the measures of the sensors with commercial motion tracking systems (e.g., Vicon, Optotrak). Such systems are capable of collecting quantitative three-dimensional (3D) kinematics across a wide range of tasks (e.g., pointing, reaching, grasping) and allow generation of kinematic measures, such as limb positions and velocities accurate to sub-millimetre level (e.g., [262]). For example, Tung et al. [164] evaluated the reliability and accuracy of LMC, to measure finger positions. Participants were instructed to perform pointing tasks and LMC data were compared to an Optotrak marker attached to the index finger. Across all trials, Root Mean Square (RMS) error of the LMC system was around 18 mm and thus considerably differs from the measures of Weichert et al. The reason for such deviating measures may be due to some weakness in the measurement technique of the LMC. A possible solution may provide a method to assess the searchable space of *Pham and Pathirana* [263]. The novelty of the approach is the combination between the inverse kinematic solver and the static and dynamic constraints of the human hand to deduce the reachable space from the motion path of the fingertip positions acquired from the LMC. This approach overcomes the weakness of the LMC and exploits the ability of accurate tracking of the fingertips using the LMC.

The evaluation of system performance is crucial because it has an effect on interface performance and user performance. For example, the frame rate probably needs to be at “real-time” levels before a user will feel present. Also, in a collaborative setting, task performance will likely be negatively affected if there is too much network delay. System performance refers to typical computer or graphics system performance, using metrics such as frame rate, latency, network delay, and optical distortion. From the interface point of view, system performance metrics are really not important in and of themselves. Rather, they are important only insofar as they affect the user’s experience or tasks. The majority of the evaluated studies is based on gestural interactions. Negative effects of recognition errors and latencies should be kept as low as possible to avoid interruptions of the natural interaction flow, namely, the responsiveness of the interaction system should be on a level which does not affect the perception and behaviour of the user [264]. In response time research, 0.1 s is agreed as the limit for having the user feel that the system is reacting instantaneously [265]. This limit will, however, not hold for the continuous interaction with virtual objects. For example, studies of flight simulators have shown that viewpoint control lags as short as 50 ms already have a significant impact on user performance [266]. In a more recent study Teather et al. [267] employed Fitts’ law to assess the effect of delays in pointing movements. Their results revealed that a delay of 40 ms causes relative performance costs (throughput) of around 15%, whereas the performance costs of a delay of 190 ms was already 50%. Thus, the real-time characteristics of the interactive systems should be especially evaluated in relation to the users task context.

## 6. Discussion

In the literature reviewed in this paper, the LMC was evaluated against competing devices and various application domains. In this section the LMC is discussed in the reviewed application domains with respect to competing devices. In the medical field, LMC was reported suitable for use as an interaction device in sterile environments. The same applies for the Kinect, however, despite the LMC providing higher accuracy, usability in the reviewed system was rated lower. For navigation, in three-dimensional anatomical models the usability of LMC was rated high. The Myo device was used as a monitoring device in physiotherapy, but due to reported delays in gesture recognition, limited use was reported as a general interaction device. However, this could be overcome with newer versions of the SDK.

Since Sign Language Recognitions (SLRs) are based on different gestures that are not limited to hands or fingers alone, a combination of the Kinect sensor for upper body poses and the LMC for finger gestures seem a promising approach for SLR system design.

In the field of application of authentication and identification, the Kinect 2.0 sensor was used to derive biometric hand features from depth and RGB images with reported detection accuracies of more than 80%. However, the reviewed works showed no information on False Acceptance Rate (FAR). Reviewed authentication approaches using LMC showed varying results of detection accuracy between 75% and 99%. It can be observed that recognition rates increased by adding user-specific gestures to systems based on biometric features.

In the context of robot (tele-)operation, simple tasks with low interaction rates, such as threading needles, were performed comfortably with the LMC. In highly interactive tasks, for example, controlling a vehicle, LMC-based interaction does not provide direct haptic feedback and was thus rated as inappropriate. In the context of drone navigation, vision-based interaction was presented for outdoor navigation tasks. In controlled indoor environments, hand gesture interaction with the LMC was reported most natural.

Gamification based applications benefited from LMC interaction as long as simple tasks like obstacle avoidance were performed and digital musical instruments designed with LMC interaction showed high usability ratings only if designed specifically for the device. Reviewed works trying to create virtual versions of keyboard or piano instruments suffered from high tracking loss rates due to occlusion issues and varying latency of the device.

Overall limitations of the LMC were reported high influences of bright light conditions and high tracking loss of up to more than 30%. The latter may be overcome by the reviewed approach using multiple Leap Motion Controlers (LMCs) to avoid self-occlusion of fingers in certain poses.

In addition to its suitability for simple interaction tasks in desktop applications, the LMC showed promising results in VR applications and execution of secondary tasks. The use of the LMC to perform secondary tasks showed a benefit for primary tasks that are heavily dependent on keyboard interaction. Secondary tasks performed with LMC reduced time on task for primary tasks. Compared to a desktop application that interacts only with LMC , a VR application with the LMC attached to a HMD showed better user performance.

In addition, the data used in the studies presented are usually not shared. An increasing availability of the research data, for example, captured sensor data, would facilitate the comparison of existing and the development of new approaches (e.g., for gesture recognition).

## 7. Conclusions

This work gave an overview of the state of the art three-dimensional HCI. Different areas of application were reviewed and proposed sets of methodologies for the definition and recognition of gestures were presented. Review of performance evaluation techniques and their realisation for the presented input devices and proposed interaction designs was given.

With upcoming output HMD devices for VR, new natural interaction devices and interaction designs are considered. This is emphasised by the vast amount of proposed interfaces for three-dimensional input devices and their evaluation. Starting with vision-based interaction using single CCD cameras or multiple cameras for three-dimensional interaction, gesture recognition techniques suffered from the same problems. Hand segmentation against different (non-static) backgrounds under varying illumination conditions prevent design of robust algorithms. This leads to the creation of application-dependent recognition or tracking approaches [155]. Depth based sensors, such as the Kinect, gave new impulses in creating gesture recognition systems. Skeletal tracking with these sensors is used in many real-world applications and Software Development Kits (SDKs) for the integration in new designed Human-Computer Interactions (HCIs) are available. However, approaches for challenging environments with low lighting or occlusions are missing [156]. Another limitation is that depth sensors as well as cameras are mostly installed in a fixed position and are employed from fixed positions. With higher accuracy and range of upcoming sensors, users could consider to move freely and thus disappear while using graphical interfaces [161].

Application independent tracking, modelling and recognition is only possible with the help of certain standards. These will further provide guidance for users and will lead to intuitive interaction without the need of specialised training sessions. For two-dimensional interaction, the WIMP metaphors form the standard for interface design. The same applies to touch-based interaction due to the widespread use of smartphones and tablets and ISO 9241-960 [268]. Due to the complexity of three-dimensional interaction tasks, up to now, no efforts in standardisation of three-dimensional interaction are taken [3]. This is documented by the vast amount of evaluation studies for the growing number of available VR systems and associated interaction devices.

It is hoped that this survey paper, by pointing out relevant methods and evaluation techniques for three-dimensional interaction, will initiate a discussion on professional standards and will serve as a useful resource to researchers involved in designing and implementing novel HCI concepts.

## Figures and Tables

**Table 1:** Overview of gesture recognition frameworks for hand detection.

Device	Application	Methods	References	Results
Myo	Gesture Recognition	Spectral CRC recognition vs. Myo-SDK recognition	[159]	97.3% accuracy
Myo & LMC		Myo-SDK gestures and LMC-SDK gestures	[54]	n/a
		LMC evaluated with optical motion capture system. EMG data compared with BioFlex EMG sensors.	[215]	n/a
	Data Fusion and Tracking	Data fusion using Kalman filter	[194]	n/a
Kinect	Gesture Recognition	Kumar et al. provide a detailed survey on Kinect based gesture recognition systems	[152]	–
	SLR	Gaussian skin colour model; LDA dimension reduction and classification	[111]	99.8%
	NUI	Thresholding and blob search	[216]	n/a
LMC	Authentication	Finger length and distance to palm; NB, RDF and NN	[146]	Acceptance rate (1% false positive): 75.78% (NB), 78.04% (RDF), 78.55% (NN)
		Normalization scheme and DTW to calculate distance between gestures	[208]	86%–91% accuracy
		LMC hand model and circle gesture, RDFC	[148]	99% static, 98% dyn. accuracy. Equal Error Rate (EER) 0.8
		LMC SDK-Hand model values; k-NN, NN, SVM, logistic regression, functional trees, logic trees	[217]	≥90% correct classified instances
	Human-Robot Interaction	Rotation gesture and grab strength; inverse kinematics	[81]	n/a
		Hand position tracking, map gestures to robot commands	[91,92,93]	n/a
		Hand tracking. Particle filter and Kalman filter	[218]	n/a
		LMC hand tracking, Tool Center Point (TCP) mapped to hand position	[84]	Tracking Error ≤ 3.32 mm
		Fingertip Positions (FPs) mapped to robot TCP	[87]	Repeatability 1 mm
	SLR	FPs, position of joints, tip velocity, pinch strength. Recognition with machine learning	[210]	72.78% (k-NN), 79.83% (SVM) recognition rate
		Multi LMC, covariance intersection and Kalman (fusion), FPs, joints HMM(recognition )	[107]	Accuracy: Multi LMC ≥ 84.68%, Single LMC ≥ 68.78%
		Leap Trainer (LeapTrainer: https://github.com/roboleary/LeapTrainer.js (accessed on 12 March 2018)) for gesture design. Motion tracking, GTM, ANN, CC for recognition 3d FPs, ANN for	[109]	52.56% (GTM), 44.87% (ANN), 35.90% (CC) accuracy
		Palm translation (phalanges to palm distance), bone translation (phalanges to next phalanges start). Classification with SVM	[112]	Palm translation 99.28%, bone translation 98.96% accuracy
Kinect & LMC		FPs and direction, palm of hand. HMM, BLSTM-NN based sequential classifiers and combination of both	[152]	Overall accuracy (97.85%, 94.55%) (single handed, double handed) combined, (97.38%, 93.64%) HMM, (87.63%, 83.49%) BLSTM-NN
	Sign Language Training and Transmission	Kinect for FaceShift and LMC to capture hand movements	[219]	n/a
		Leap Trainer for gesture, pose learning and recognition	[141]	n/a
LMC	Surgery Training	Speed, acceleration, smoothness, distance between hands	[73]	Tracking loss 31.9%
		Track positions of instrument over LMC	[220,221]	static precision ≤ 2.5 mm, dynamic ≥ 2 mm ≤ 15 mm
	NUI (VR)	Hand gesture interface based on LMC-SDK	[69,105,118,121,123,127,139,170,171,172,173,174]	n/a
	NUI (Desktop)	Hand gesture interface based on LMC-SDK	[53,55,56,93,120,124,126,128,133,137,168,175,176,177,178,179,180,181,182,183,184,185,186,187,188,189,190]	n/a
	Rehabilitation	LMC hand tracking, UNITY, evaluation against Novint Falcon	[66]	device evaluation
		Joints of fingers, angles between them	[58]	error: ≥ 2.5° ≤ 9.02°
		FPs, direction of forearm and hand, palm normal, joint angle of wrist and knuckles, static. Decision-tree, k-NN, and SVM classification	[67]	Re substitution error: Decision-tree ≤ 23.04%, k-NN ≤ 0.49%, SVM ≤ 2.1%
		FPs, roll, pitch, yaw Fast Fourier Transform (FFT)	[222]	feasibility study
		Generate hand-model of FPs direction vectors (inverse kinematics)	[223]	tracking issues
		LMC hand tracking and gestures	[70,200,224,225]	n/a
		Palm tracking, distance between FPs and palm, angle between fingertip vector and vector from wrist to palm. LDA, SVM, CRF, HMM and combinations for classification	[211]	SVM 88.44%, LDA 87.67%, SVM+CRF 98.74%, LDA+CRF 99.42%, SVM+HMM 98.56%, LDA+HMM 98.96%
	Rehabilitation / Fusion	Multi LMC, motion tracking; Iterative Closest Point (ICP)	[63]	n/a
	Prefrontal Cortex Activation (Immersive Environments)	LMC-SDK hand orientation and FPs, 20 channels FNIRS, heart rate; Analysis of Variance (ANOVA)	[226]	user experiment
	Gesture Recognition	Distance of FPs to palm; comparing to reference vector in database	[227]	Accuracy with Cosine similarity metric 90%, Euclidean 88.22%, Jaccard 86%, dice similarity 83.11%
		FPs ANN	[79]	accuracy ≥ 70.52% ≤ 87.6%
		FPs, scikit-learn (scikit-learn, http://scikit-learn.org/stable/ (accessed on 12 March 2018)) (SVM)	[228]	Accuracy: ≥ 75%
		Palm direction, palm normal, FPs, palm centre. HCNF classifier	[212]	Two datasets: 95% and 89.5% accuracy
		FPs tracking. Built-in gestures	[213]	accuracy at 400 lx and 800 lx dynamic 78%, static 90%, > 1000 lx: dynamic 2%, static 85%
		Motion tracking, Convolutional Neural Network (CNN) and time series recognition with HMMs for gesture detection	[229]	CNN 92.4%, HMMs 50% for time series
		Distance between palm centre and fingertips, k-NN, Multi Layer Perceptron (MLP), Multinomial Logistic Regression (MLR) classification (static)	[230]	k-NN ≥ 70% ≤ 95%, MLP 70% ≤ 90%, MLR 85% ≤ 90%
		LMC hand tracking, threshold-based gestures	[138]	≥93%
		Grammar of air gestures	[201]	Extended Backus-Naur
		LMC-SDK skeletal tracking	[231]	Mathematical model of hand occlusion
		Centre position of hand, Recurrent Neural Network (RNN) classification	[232]	Recognition rate ≥ 77%
	Hardware Design	LMC on top of mouse device. Built-in gestures	[233]	Hardware design, user experiment
	Multiple Leap Motions	Sum of angles of first three joints and the lateral movement angle of each finger; Self-Calibration	[234]	simple kinematic model of finger
